# Future horizons in diabetic macular edema management: nanocarriers as a promising alternative to conventional anti-VEGF therapy

**DOI:** 10.1097/MS9.0000000000004300

**Published:** 2025-11-17

**Authors:** Rabia Ammer, Meher Wali Khan, Muhammad Hanan, Muhammad Talha, Mahnoor Fatima

**Affiliations:** a Department of Medicine, University of Faisalabad, Faisalabad, Pakistan; b Department of Ophthalmology, Hamad Medical Corporation, Doha, Qatar; c Department of Medicine, King Edward Medical University, Lahore, Pakistan; d Department of Medicine, Shaheed Ziaur Rahman Medical College, Rajshahi Medical University, Bogura, Bangladesh

*Dear Editor*,

Diabetes mellitus is a major 21st-century health epidemic, with severe sight-threatening ocular manifestations^[1]^. Prolonged hyperglycemia substantially increases the risk of developing diabetic retinopathy (DR), a debilitating microvascular complication, and affects nearly all type 1 and over 60% of type 2 diabetes patients within two decades^[1,2]^. The most vision-threatening complication of DR is diabetic macular edema (DME), characterized by fluid accumulation in the macula resulting from the breakdown of the blood–retinal barrier and vascular hyperpermeability^[3]^. This complex pathogenic process is driven by angiogenesis and mediated primarily by vascular endothelial growth factor (VEGF), which disrupts tight junctions in retinal endothelial cells, leading to progressive fluid accumulation and potential blindness^[1]^. This is in line with the TITAN Guidelines on the need for transparency in AI use in healthcare^[4]^.

DME stands as the most common cause of visual impairment, with a global prevalence affecting nearly 7% of individuals with diabetes^[5]^. It affects approximately 75 000 new US patients annually, and 2–8.2% diabetic patients develop it within 5 years of diagnosis, rising to 28% after 20 years^[3,5]^. Intravitreal administration of anti-VEGF agents, such as ranibizumab or bevacizumab, is currently recognized as the first-line treatment^[6]^. These inhibitors effectively suppress neovascularization and reduce vascular permeability^[2]^. Despite their efficacy, conventional anti-VEGF therapy requires frequent monthly intravitreal injections due to the drugs’ short half-lives, structural instability, and protease susceptibility, leading to high patient burden and poor compliance^[1,2,7]^. The invasive procedure risks serious complications, including endophthalmitis, hemorrhage, and retinal detachment^[2,7]^. Systemic side effects such as hypertension and proteinuria associated with agents like bevacizumab present additional concerns^[1,2]^.

Nanotechnology has emerged as a transformative solution to the limitations of conventional treatment, offering innovative strategies to improve patient compliance and therapeutic outcomes^[7]^. Nanocarriers function as specialized protective vehicles that encapsulate drugs, shielding them from degradation and elimination while prolonging circulation time and enhancing stability^[2]^. Compared to traditional formulations, nano-preparations provide multiple advantages, including the ability to cross biological barriers due to their small particle size, targeted delivery to specific sites of action, sustained-release properties that extend therapeutic duration, and protection from enzymatic degradation. For instance, encapsulating bevacizumab in mesoporous silica nanoparticles extends its release up to 28 days *in vitro* and results in a longer mean retention time *in vivo*^[1]^.

Despite their potential, the clinical translation of nanoparticles for DME faces significant challenges. These include intricate formulation and manufacturing processes, potential immunogenic responses or toxicities associated with the carrier materials, and difficulties in scaling up production for widespread clinical use. Furthermore, their pharmacokinetic profile, particularly clearance rates from the vitreous, exhibits high variability contingent upon nanoparticle characteristics such as size and surface chemistry^[2]^.

The future of nanotechnology in ophthalmology is promising, particularly in the treatment of DME. Advances in biocompatible and degradable nanoparticles enhance safety, drug delivery efficiency, and therapeutic durability while reducing systemic side effects. Smart nanocarriers offer improved retinal penetration, prolonged drug residence, and combination therapy options, addressing limitations of current regimens. Future progress depends on overcoming regulatory and clinical challenges, and randomized controlled trials are essential to establish efficacy against existing therapies.

Nanotechnology, with its vast features, has a promising future in the field of ophthalmology, especially in the treatment of DME. Now there is enhanced safety, more efficient delivery of the drug, and enhanced duration by the use of degradable and biocompatible nanoparticles, all with little systemic side effects. By the use of smart non-carrier, there is enhanced retinal tissue penetration, prolonged duration of action, and an option of a combination therapy approach, efficiently solving the issues that are with current therapy. However, to truly prove its superiority over existing therapies, there is a need to bypass regulatory and clinical challenges, and randomized controlled trials are also essential.

## Data Availability

Not applicable to this study.
